# Examining Relationships Between Vitamin D Deficiency, Healthcare Access, Hispanic Ethnicity, and General Health Rating among Aging Rural West Texans: A Project FRONTIER Study

**DOI:** 10.1002/alz.095410

**Published:** 2025-01-09

**Authors:** Claudia Morris, Riley McCready, Grace McCrea, Boris Decourt, J. Josh Lawrence

**Affiliations:** ^1^ Texas Tech University Health Sciences Center, Lubbock, TX USA

## Abstract

**Background:**

Vitamin D (VD) deficiency is a risk factor for dementia and Alzheimer’s Disease (AD). Previously, we described health disparities in VD status, depression, and Hispanic ethnicity (HE) in an aging West Texas population from Project FRONTIER (Facing Rural Obstacles to Health Care Now Through Intervention, Education, and Research). Using the same sample, we examined relationships between VD status, health care access, and General Health Rating (GHR)

**Method:**

Of 299 participants in which serum 25‐hydroxyvitamin‐D levels were available, we examined relationships between access to care, VD, HE, and GHR. Logistic and linear regression analyses were performed on binary and categorical variables, respectively. We used Mann Whitney U tests for between group comparisons.

**Result:**

A significant negative association was found between the probability of health insurance and VD level (p = 0.0042). Lower VD levels were observed in uninsured (24.9 ± 1.4 ng/ml) compared to insured (29.4 ± 0.8 ng/ml) participants (p = 0.004). We found a significant negative association between VD levels and the probability of experiencing a time in the past 12 months when cost prevented seeing a doctor (n = 294, p<0.0001). We found a significant negative association between VD level and the length of time it had been since the participant had seen a doctor (p<0.0001). We found a significant negative correlation between VD level and GHR (p<0.0001). Lower VD levels were observed in HE (21.40 ng/ml) compared to non‐HE (34.00 ng/ml) participants (p<0.0001). Finally, we found a significant negative correlation between HE status and GHR (p<0.0001).

**Conclusion:**

Our results reveal that insurance level, access to care, and length of time since seeing a physician likely impact VD status in this aging population. Additionally, our results reveal that VD status is negatively correlated with HE. Our results reveal that HE status and VD status likely impact GHR. Finally, the data highlights areas of healthcare in rural West Texas needing improvement, especially considering that studies show correlations between Vitamin D deficiency, dementia, and AD.

